# *Candida* Bezoars in Adults: Determining Optimal Management

**DOI:** 10.1089/cren.2017.0021

**Published:** 2017-04-01

**Authors:** Matthew A. Rohloff, Jaschar Shakuri-Rad, Alexander P. Dehaan

**Affiliations:** Metro Health Hospital—University of Michigan Health, Wyoming, Michigan.

**Keywords:** fungal bezoar, fungal ball, *Candida* funguria, percutaneous nephrostomy, amphotericin B instillations

## Abstract

Fungal bezoars, or fungal balls, are rare pathologic consequences of funguria in immunocompromised patients. Current treatment recommendations are based on expert opinion and low level evidence. We present a case of a *Candida glabrata* bezoar that was effectively treated with percutaneous amphotericin B instillations. A subsequent literature review is presented to assess the available case reports and treatment outcomes of *Candida* spp. bezoars in adults.

## Introduction

There are a scatter of case reports of fungal bezoars in the adult urologic literature and even fewer caused by *Candida glabrata*. As a result of the paucity of data, there are no guidelines outlining a treatment algorithm for management of *Candida* bezoars. The Infectious Disease Society of America 2016 guidelines recommend surgical removal, antifungal treatment, or irrigation of amphotericin B through a nephrostomy tube. These recommendations are based on low-quality evidence.^1^ The purpose of this case report is to discuss a patient with a *C. glabrata* bezoar who was effectively managed with intravenous antifungals and percutaneous nephrostomy tube instillations of amphotericin B.

## Case Report

A 56-year-old female with a medical history significant for type 2 diabetes mellitus, chronic pancreatitis, and alcoholism was admitted to our institution in October 2016 with the chief complaint of abdominal distention and generalized malaise. Review of systems was pertinent for mid-back pain that radiated to her bilateral lower extremities, dysuria, incomplete voiding, and urinary urgency. The patient did not have any urologic history.

**FIG. 1.**
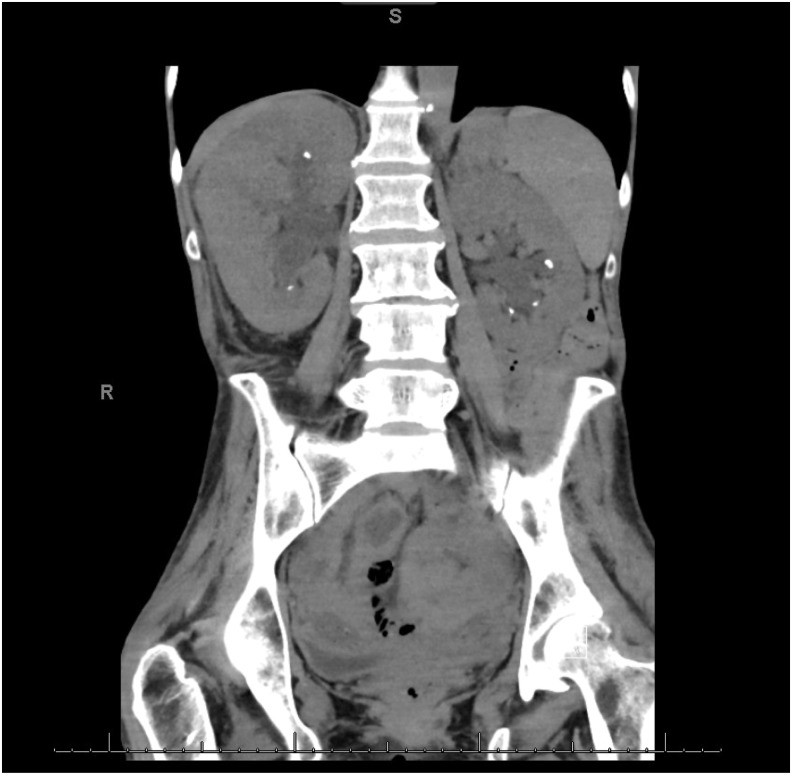
CT of abdomen and pelvis. Multiple bilateral renal stones measuring between 1 and 4 mm. Bilateral pelvocaliectasis. No discrete renal lesions although renal fungal ball cannot be excluded.

Laboratories were significant for leukocytosis of 11.0 K/μL, creatinine of 2.275 cc/dL, GFR of 22 cc/min/1.73 m^2^, and a urinalysis that showed large RBCs, WBCs, and many yeast cells. Noncontrast CT of abdomen and pelvis was obtained that showed multiple bilateral nonobstructing renal stones, but no discrete intrarenal lesion (Fig. 1). The patient was found to have a postvoid residual >999 cc and was straight catheterized for 3000 cc of urine.

**FIG. 2.**
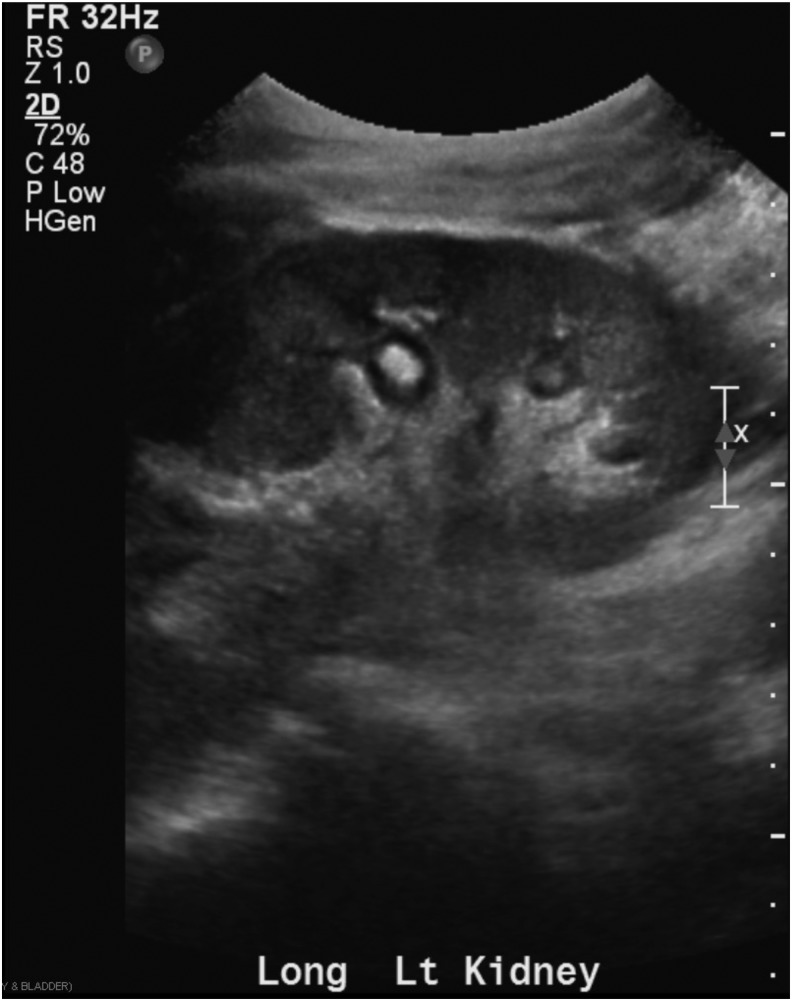
Renal ultrasonography. Seven millimeter, left sided interpolar nonshadowing hyperechoic foci in the renal collecting system.

Blood and urine cultures yielded *C. glabrata.* A fungal ball remained high on the differential diagnosis, and despite an equivocal CT scan, a renal ultrasonography was obtained. The left kidney showed a 7 mm echogenic structure resembling a fungal ball (Fig. 2). The infectious disease team placed the patient on oral micafungin and IV diflucan. Systemic amphotericin B was avoided to prevent exacerbation of kidney injury in a patient with compromised renal function.

The patient also underwent percutaneous nephrostomy tube placement by interventional radiology to facilitate amphotericin B irrigations. She continued with daily 50 mg instillations of amphotericin B in 500 cc of water for 6 days until resolution of sepsis. The patient continued to improve and it was determined that further operative intervention was unnecessary. She remained on IV micafungin and diflucan for a total of 14 days. On discharge, blood and urine cultures showed no fungal growth and the patient's leukocytosis and acute kidney injury had completely resolved. The patient was last seen 3 months posthospitalization and remains asymptomatic.

## Discussion and Literature Review

For the past decade, there has been a 300% increase in the prevalence of opportunistic fungal urinary tract infections (UTIs).^2,3^ It is estimated that 5% of urine cultures yield *Candida* spp. and 26.5% of UTIs with indwelling Foley catheters are inoculated with fungi.^2^ Although asymptomatic funguria requires no treatment, symptomatic funguria in the setting of underlying immunosuppression can lead to significant pathology analysis. This is further exacerbated by patients inoculated with *C. glabrata,* as they are often resistant to azole antifungal agents and require systemic or local amphotericin B irrigation.^4^

Historically, treatment of fungal bezoars has been based on clinical presentation and physician discretion. Recently, the Infectious Disease Society of America 2016 guidelines were presented to help assist in management decisions. The recommendation to utilize surgical removal has been described as “central to effective treatment,” but is based on two small case reports and low-quality evidence.^1^ In an attempt to better categorize treatment modalities and outcomes, case reports involving renal candidiasis and fungal balls in adults were obtained utilizing the database from National Center for Biotechnology Information (NCBI) and U.S. National Library of Medicine (NLM) (Table 1).

**Table 1.** Comprehensive Literature Review of Available Case Reports of *Candida spp*. Fungal Bezoars

*Author*	*Age/sex*	*Comorbid conditions*	*Microbiology*	*Treatment*	*Outcome*
Gerle^5^	17 M	DM, quadriplegia	*Candida albicans*	Bilateral nephrostomies with endoscopic removal	Death 2 months after admission
Gerle^5^	59 F	DM, quadriplegia	*C. albicans*	Retrograde stent placement with amphotericin irrigations	Discharged home with no complications and no reported follow-up
Turner et al.^6^	62 F	DM, left nephrectomy for stone disease	*C. albicans*	Nephrolithotomy and flurocytosine	Discharged home with no complications 1 month postdischarge
Olivero et al.^7^	22 M	IV drug abuser	*Candida* spp.	No treatment—spontaneous expulsion	Discharged home with no complications 6 months postdischarge
Ireton et al.^8^	49 F	Renal transplantation, renal calculi	*C. albicans*	Nephrostomy with amphotericin instillations	Discharged home with no complications 6 months postdischarge
Doemeny et al.^9^	65 F	DM	*Candida glabrata*	Amphotericin B	Discharged home with no complications, no reported follow-up
				instillations retrograde, percutaneous extraction	
Franco et al.^10^	NR F	DM, renal transplantation, and immunosuppressive agents, prophylactic ureteral stent	*C. albicans*	Oral ketoconazole	Discharged home with no complications and 12 months postdischarge
Keung et al.^11^	61 F	Follicular cell lymphoma, bone marrow transplantation, chemotherapy	*C. albicans*	Systemic amphotericin B and fluconazole	Discharged home with no complications and no reported follow-up
Praz et al.^12^	63 M	Hepatic transplantation with fungal peritonitis	*Candida tropicalis*	Bilateral percutaneous nephrostomy tubes	Unreported
				Local/systemic fluconazole	
Praz et al.^12^	84 M	DM, neurogenic bladder, bladder diverticulectomy for stones	*C. albicans*	Percutaneous nephrostomy tube	Discharged home with no complications, no reported follow-up
				Cystotomy with removal of fungus ball	
Onozawa et al.^13^	61 M	DM, alcoholic cirrhosis, candida endopthalmitis	*C. albicans*	Fluconazole	Discharged home with no complications and no relapse for 3 years.
Berlanga et al.^14^	57 F	DM, emphysematous pyelonephritis, right ureteral stent (2/2 pyelonephritis)	*C. glabrata*	PCN tube with amphotericin B, systemic amphotericin with endoscopic removal	Discharged home with no complications and no reported recurrence 3 weeks postdischarge
Jegannathan et al.^15^	45 M	DM, ulcerative colitis	*C. albicans*	Radical nephrectomy	Discharged home with no complications, no reported follow-up
Levin et al.^16^	50 F	Graves' disease, nephrolithiasis	*C. albicans*	Nephrostomy tube with amphotericin B instillations, radical nephrectomy	Discharged home with no complications 3 weeks postdischarge
Rohloff et al.	56 F	DM, alcoholism	*C. glabrata*	Nephrostomy tube with amphotericin B instillations, micafungin and diflucan	Discharged home with no complications 3 months postdischarge

F, female; DM, diabetes mellitus; M, male; NR, not reported.

Comprehensive literature review of 15 case reports yielded patients who were effectively treated both operatively and nonoperatively. Seven patients of the 15 operative case reports chose operative removal, which consisted of two nephrectomies, one cystotomy, one pyelolithotomy, and three endoscopic retrievals. Of the seven reported operative cases, six patients were discharged home in stable condition and one died before discharge. The other eight cases were treated with conservative approaches utilizing local antifungals, systemic antifungals, or spontaneous expulsion. Seven of the eight cases were discharged to home in stable condition and the outcome of one of the cases was unreported.

**FIG. 3.**
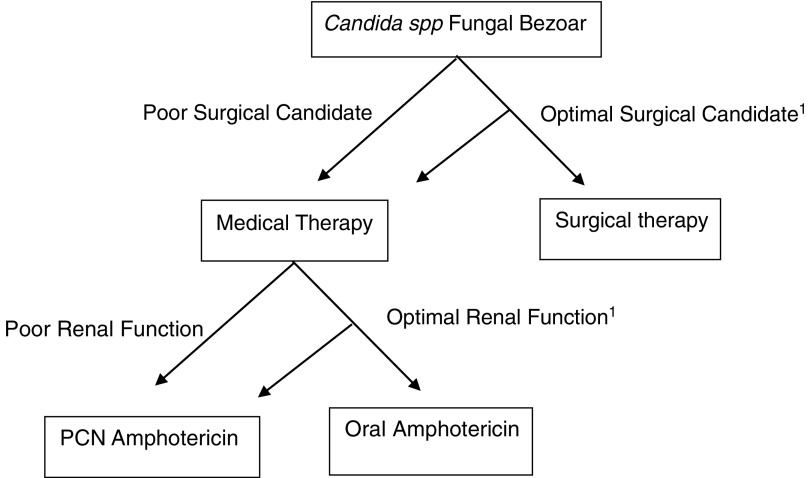
Treatment algorithm for patients with *Candida* spp. fungal bezoars. Literature review shows that medical therapy is not inferior to surgical management. 1, dependent on physician discretion; PCN, percutaneous nephrostomy.

Although strong evidence-based suggestions on superior therapy cannot be obtained from a few case reports, it is evident that both modalities have had advantageous outcomes. Operative intervention, although potentially curative, is not without its own inherent risks. Based on our patient, and the findings of seven other medically managed cases, it is reasonable to suggest that medical management of fungal bezoars is not inferior to operative intervention. Optimal treatment regimens for individual patients are ultimately based on the discretion of the treating physician. Figure 3 shows an algorithm to further assist with the treatment of *Candida* bezoars.

## Conclusions

Although C*andida* bezoars are extremely rare in the urologic literature, it is imperative that recommendations based on quality data are obtained to optimize treatment. The presented case is a 56-year-old female with a *C. glabrata* bezoar who was effectively treated with systemic antifungals and percutaneous amphotericin B instillations. This case serves as a reminder that fungal bezoars can be managed without fungal ball extraction and still have advantageous outcomes.

## Abbreviations Used

CT: computed tomography
DM: diabetes mellitus
GFR: glomerular filtration rate
IV: intravenous
NCBI: National Center for Biotechnology Information
NLM: U.S. National Library of Medicine
NR: not reported
UTI: urinary tract infection
